# Supplementary material for: Causal Relationship between Aging and Anorexia Nervosa: A White-Matter-Microstructure-Mediated Mendelian Randomization Analysis
Source: Biomedicines. 2024 Aug 16;12(8):1874. doi: 10.3390/biomedicines12081874 (PMC11351342; doi:10.3390/biomedicines12081874)
Supplement: Supplementary file 1 [file biomedicines-12-01874-s001.zip › Supplementary Figure/Figure S8/Leaveoneout plot of MR analysis for TL to ubm-b-2047.pdf]

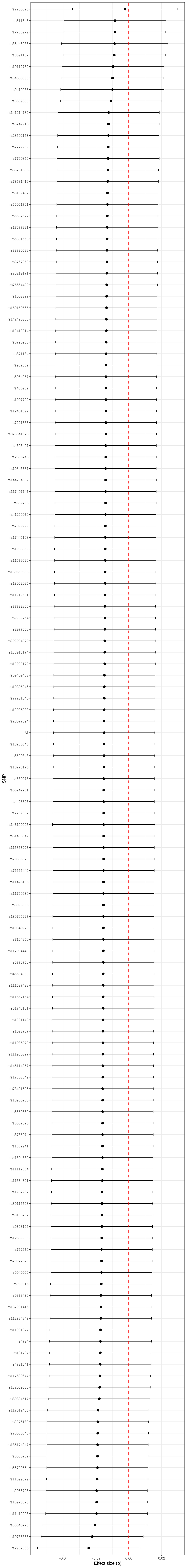
SNP
rs7705526
rs611646
rs2763979
rs35446936
rs3891167
rs10112752
rs34550383
rs9419958
rs6669563
rs141214782
rs5742915
rs28502153
rs7772289
rs7790856
rs66731853
rs73581419
rs8102497
rs56061761
rs6587577
rs17677991
rs6881568
rs73730598
rs3767952
rs76219171
rs75664430
rs1003322
rs150150565
rs142426306
rs12412214
rs6790988
rs871134
rs932002
rs6054257
rs450962
rs1907702
rs12451892
rs7221585
rs376641875
rs4695407
rs2538745
rs10845387
rs144204502
rs117407747
rs869785
rs41269079
rs7099229
rs17445108
rs1985369
rs11579626
rs139669835
rs13062095
rs11212631
rs77732866
rs2282764
rs2977608
rs202034370
rs188918174
rs12932179
rs59409453
rs10805346
rs77231040
rs12925933
rs28577594
A8
rs13230646
rs6590343
rs10773176
rs4530278
rs55747751
rs4498805
rs7209057
rs143190905
rs61405042
rs116863223
rs28363070
rs76666449
rs11426156
rs11769630
rs3093888
rs139795227
rs10840270
rs7164950
rs117034449
rs6776756
rs45604339
rs111527438
rs11557154
rs61748181
rs1291143
rs1023767
rs11085072
rs111950327
rs145114957
rs17803849
rs78491606
rs10905255
rs6659669
rs6007020
rs3785074
rs1332941
rs41304832
rs11117354
rs11584821
rs1957937
rs80116508
rs8105767
rs9398196
rs12369950
rs762679
rs79977579
rs9940099
rs939916
rs9878436
rs137901416
rs112394943
rs11991877
rs4724
rs131797
rs4731541
rs117630647
rs182059586
rs80324517
rs117512405
rs2276182
rs76065543
rs185174247
rs6536702
rs56799554
rs11699829
rs2056726
rs16978028
rs11412296
rs35640778
rs10768683
rs2967355
-0.04
-0.02
0.00
0.02
Effect size (b)
